# Recurrent Stroke in a Child with TRMA Syndrome and SLC19A2 Gene Mutation

**Published:** 2018

**Authors:** Parvaneh KARIMZADEH, Toktam MOOSAVIAN, Hamidreza MOOSAVIAN

**Affiliations:** 1 Pediatric Neurology Research Center, Research Institute for Children Health, Shahid Beheshti University of Medical Sciences, Tehran, Iran.; 2 Pediatric Neurology Department, Mofid Children’s Hospital, Faculty of Medicine, Shahid Beheshti University of Medical Sciences, Tehran, Iran.; 3 Department of Clinical Pathology, Faculty of Veterinary Medicine, University of Tehran, Tehran, Iran.

**Keywords:** Thiamine-responsive megaloblastic anemia (TRMA syndrome), Stroke, Infant

## Abstract

Here we report a 5-month-old boy with thiamine Responsive Megaloblastic Anemia syndrome (TRMA syndrome) with several attacks of stroke, admitted to Mofid Children's Hospital, Tehran, Iran, in 2016. In addition to the cardinal clinical manifestations of the syndrome, other manifestations comprise thiamine-responsive megaloblastic anemia, diabetes mellitus, and sensor neural hearing loss. The patient showed the ischemic attack of stroke. Megaloblastic anemia and diabetes were diagnosed at 8 months and was successfully treated with vitamin and insulin prescription. After treatment of thiamine, diabetes was controlled and insulin was discontinued. In spite of the thiamine administration, the second stroke as hemorrhagic stroke occurred in the patient after a few months. TRAMA is inherited in an autosomal recessive manner. TRMA was confirmed by mutation in SLC19A2. A homozygous splice site variant was detected in SLC19A2 gene. Stroke was not reported in this syndrome (only in one report about one attack in an adult patient) but in this patient, several attacks of stroke were reported before and after thiamin administration.

## Introduction

Thiamine-Responsive Megaloblastic Anemia syndrome (TRAMA syndrome) also known as ROGER syndrome is inherited in an autosomal recessive manner. Cardinal clinical manifestations of the syndrome include thiamine-responsive megaloblastic anemia, diabetes mellitus, and sensor neural deafness (1, 2). These symptoms do not appear in all of patient's in distinct time, or all three manifests coincident (2). Additional findings include optic atrophy (3, 4), congenital heart disease (5), stroke (6), and short stature (3, 4). 

A splice site variant has detected in SLC19A2 gene locate on 1q23.3. It codes a protein that is necessary for thiamin uptake by cell (2). Mostly patient was homozygous for mutation in this gene but was described four patients with compound heterozygous mutation and novel mutation with the same symptom and response to thiamin (2). Reports revealed only one case with TRMA with stroke that she consumed oral contraceptives for ovarian cyst but she respond to thiamin therapy (6); however, our patient did not respond to thiamin well and he had stroke in spite of taking 300 mg of thiamin. Nevertheless, anemia and diabetes could be managed with this treatment.

Here we report a 5-month-old boy with TRMA syndrome with several attacks of stroke.

## Case presentation

This study was conducted on a 5 months-old boy with focal seizure history, admitted to Mofid Children's Hospital of Tehran, Iran in 2016. Declaration of informed consent was obtained from his parents. He was product of consanguineous marriage. The history of detecting of neurodevelopmental milestones was in normal limits. Brain magnetic resonance imaging (MRI) revealed hemorrhagic stroke in right Middle Cerebral Artery (MCA) region. Initially, the seizures were controlled by antiepileptic drugs. In addition, the patient showed left-sided hemiplegia, regression in his neurodevelopmental milestones, and cognitive impairment. 

Results for complete lab tests for evaluation of stroke including, serum amino acid chromatography, antiphospholipid antibody, factor V Leiden and G20210A prothrombin, were not significant for collagen vascular disease, thrombi formation, and other disorders. Three months later, the patient was presented with lethargy. More evaluation showed macrocytic anemia (MCV> 95Hb =3/5), insulin-resistant diabetes (serum glucose> 400 mg/dl), and bilateral sensor neural deafness. Based on the clinical and paraclinical findings TRMA syndrome was established for the patient and additional management included thiamine was started. Two months later (in spite of thiamine administration) the patient showed right-sided focal seizure. 

**Fig 1:**
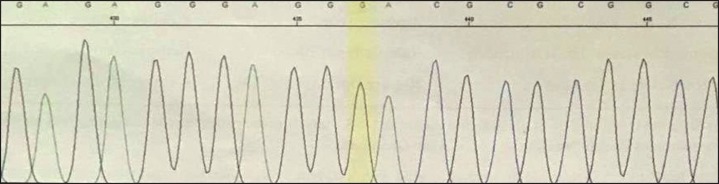
Homozygosity in the patient is shown for the detected variant in SLC19A2 gene (c.204+2T>G

**Fig 2:**
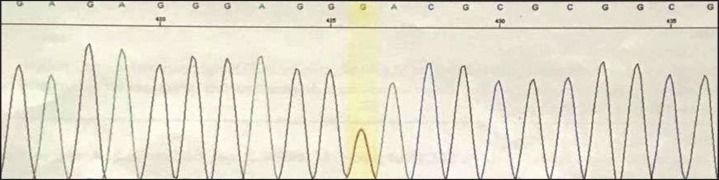
Heterozygosity in his father is shown for the detected variant in SLC19A2 gene (c.204+2T>G

**Fig 3.**
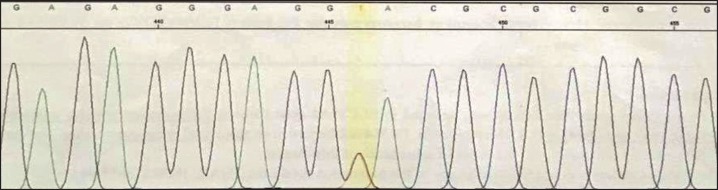
Heterozygosity in his mother is shown for the detected variant in SLC19A2 gene (c.204+2T>G

**Fig 4:**
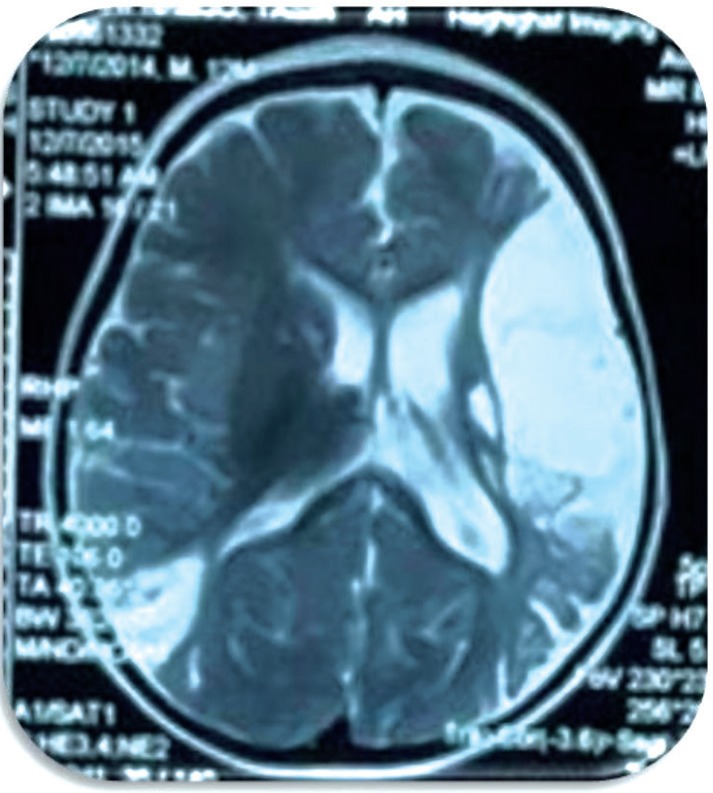
T2 Sequencing shows hypersignal changes in left temporoparietal region

**Fig 5:**
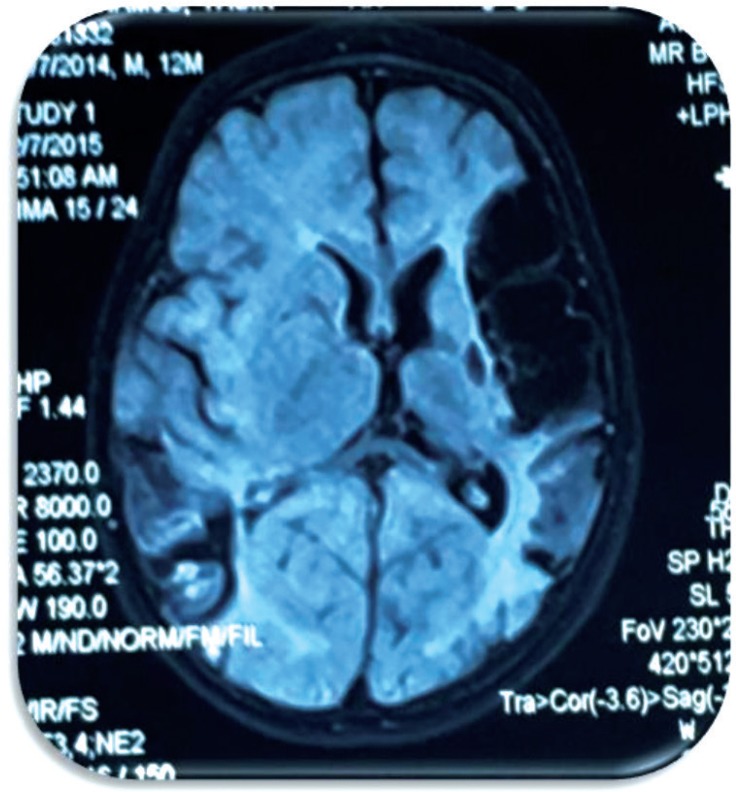
FLAIR sequencing shows hypointensity in left temporoparietal region

Now the patient is about 2.5 yr old with bilateral optic atrophy, mild bilateral hemiplegia, and cognitive disorder. He is treated by antiepileptic drugs and 300 mg thiamine per day. The last hematology and biochemical analysis showed normal blood sugar, normal lipid profile, and the hemoglobin concentration and MCV were 10 mg/dl and 75 fl, respectively. Gene analysis confirmed a homozygous splice in SLC19A2 gene (Figure 1). The detected variant c.204+2T>G on SLC19A2 gene was validated in homozygous state in the patient. The genotypes of parents were heterozygote for this variant (Figure 2, 3). TRAMA is inherited in an autosomal recessive manner, and so the genotype of the patient was consistent with the mode of the inheritance of this disease. The detected variant was not reported in any of the population databases. MRI revealed left ischemic stroke in left MCA (Figure 4, 5). Enoxaparin sodium was prescribed for two months, but his parents stopped taking his medicine.

Figure 4 & 5 shows Axial brain MRI in FLAIR & T2W sequence shows cystic encephalomalacia with peripheral gliosis in left MCA territory and right posterior watershed zone at parieto-occipital lobe consistent with remote stroke

## Discussion

TRMA syndrome is an autosomal recessive disorder caused by mutations in the SLC19A2 gene encoding the high-affinity thiamine transporter 1 (h-THTR1). Clinically, this syndrome is characterized by megaloblastic anemia, non-type I diabetes mellitus, and sensorineural deafness. In addition to the main clinical trial, some other clinical findings, such as abnormalities of the retina and optic nerve, congenital heart defects, short stature, and strokes have been described in a few cases. Most of patients were born from consanguineous parents (1, 7-10). In the present study, the patient’s parents were consanguineous. 

Typically, the current first line of therapy for patients with TRMA syndrome is pharmacological doses of thiamine (25-75 mg/d). Anemia and diabetes improve significantly with this treatment, although the erythrocytes may remain macrocytic. However, sensorineural hearing loss is apparently irreversible (2). In addition, some other clinical signs such as short stature seen in few cases may do not improve with thiamine administration (11).

In the present study, initially, the patient was presented with a history of focal seizure, and brain MRI results showed hemorrhagic stroke in right MCA region. Three months later the patient showed megaloblastic anemia, diabetes, and bilateral sensorineural deafness. Therefore, TRMA syndrome was established for the patient and additional management included thiamine repletion. Gradually macrocytic anemia and diabetes improved and MCV was decreased. In spite of thiamine administration, second stroke occurred. 

Strokes (or stroke-like episodes) are certain features of TRMA described in a few cases, even with homozygous null mutations (2). There is not any report of recurrent stroke after thiamine repletion. However, in the present study, the patient had stroke both before and after thiamine administration. 

TRMA is a genetic disorder caused by mutations in SLC19A2 on chromosome 1q23.3, that encodes a thiamine transporter protein. The disease can manifest at any time between infancy and adolescence. The majority of the mutations are null mutations because of nonsense or frameshift mutations. More than 28 mutations in SLC19A2 gene as heterozygote or homozygous have been reported leading to TRMA (2).

In the present study, molecular analyses identified a novel homozygous mutation c.204+2T>G on SLC19A2 as the cause of the disease. A homozygous nonsense mutation c.697C>T (p.Gln233*) was described in Persian families with thiamine-responsive megaloblastic anemia, however, this mutation was previously reported in a Turkish patient, as well (11). 

**In conclusion, **more studies are needed to elucidate any association between TRMA and stroke as an outcome in this disease. The patient with stroke as a clinical manifestation did not respond to treatment very well.
